# Fusarium Causing Necrotizing Myositis and Tibial Periosteitis in an Immunocompetent Patient

**DOI:** 10.7759/cureus.103705

**Published:** 2026-02-16

**Authors:** Imran Samnani, Zain Samnani, Andrew J Shychuk

**Affiliations:** 1 Internal Medicine, Veterans Affairs Medical Center, Gainesville, USA; 2 Biology, Florida State University, Tallahassee, USA; 3 Internal Medicine, University of Florida College of Medicine, Gainesville, USA

**Keywords:** chronic osteitis, fusarium, fusarium species, fusarium spp, necrotizing myositis, osteitis, osteomyelitis

## Abstract

*Fusarium* species are filamentous fungi that are ubiquitous in the environment, residing mainly in soil and associated with plants. While primarily plant pathogens, there are over 1000 subspecies (ssp) of *Fusarium*, and the more pathogenic ssp have been responsible for billions of dollars in economic losses associated with maize, rice, and wheat crops. *Fusarium* ssp have been linked to human pathogenicity, with invasive fungal infections occurring mainly in patients with hematologic malignancies, specifically leukemia. Infections with *Fusarium* spp are uncommon in immunocompetent patients, but invasive infections such as osteomyelitis are even rarer. We present a case of necrotizing myositis and tibial periosteitis in an otherwise healthy patient in the fourth decade of life. After initial diagnostic challenges and worsening condition on broad-spectrum antimicrobials tailored to bacterial pathogens, *Fusarium* was diagnosed by intraoperative cultures and histopathology. As such, antifungal coverage with amphotericin B and voriconazole was added, resulting in substantial improvement of the infection. The patient was eventually transitioned to oral voriconazole with an anticipated six to eight week course and close follow-up. At the time of preparing this report, the patient has been followed in the outpatient setting and continues to show significant improvement.

## Introduction

*Fusarium* species, filamentous fungi, are widespread in the environment and are found predominantly in soil. Although they are primarily recognized as plant pathogens capable of causing severe damage to maize, rice, and wheat crops, they have also been linked to both localized and invasive human infections, particularly as opportunistic infections in immunocompromised individuals. Invasive infections caused by *Fusarium* species have been well documented in patients with malignancies, especially hematologic malignancies such as leukemia [1-5], but only isolated cases have been described in immunocompetent hosts. Moreover, many individuals classified as immunocompetent may in fact be functionally immunocompromised, given the presence of diabetes as a potential contributory comorbidity leading to *Fusarium*-related osteomyelitis [6-7]. In other instances, infection in otherwise healthy hosts has been either posttraumatic, with a retained foreign body resulting in tibial osteomyelitis [8-9], or confined to more limited cutaneous involvement [1]. 

Other reported localized manifestations of *Fusarium* infections include fungal keratitis, cutaneous plaques or ulcers [1], subcutaneous nodules, and onychomycosis [10]. These presentations are usually associated with prior exposure to soil or plant material, a preceding traumatic event in cases of corneal involvement, and underlying nail or dermatologic disorders. The clinical manifestations of invasive *Fusarium* infection include refractory neutropenic fever, bloodstream infection confirmed by positive blood cultures, and disseminated lesions involving the lungs and skin, often presenting as tender cutaneous nodules. The mortality rate associated with invasive *Fusarium* infections ranges from approximately 50-80% percent in patients with persistent neutropenia. *Fusarium* species exhibit intrinsic resistance to multiple antifungal agents, including echinocandins, and demonstrate elevated minimum inhibitory concentrations against certain azoles, such as fluconazole and itraconazole. Voriconazole and amphotericin B are generally regarded as first-line treatment options because of their comparatively low minimum inhibitory concentrations against *Fusarium* species [11-14]. The prognosis of *Fusarium* infection correlates closely with the severity of neutropenia and the extent of immunosuppression, particularly in patients receiving immunosuppressive therapy following hematopoietic stem cell transplant (HSCT).

To our knowledge, only a single case of visceral disseminated *Fusarium solani* has been reported in a non-immunocompromised adult male. Although *Fusarium* infections are generally rare in immunocompetent individuals, they may be underrecognized due to limited data on risk factors that predispose otherwise healthy individuals, potentially resulting in delayed identification when *Fusarium* is the causative pathogen. Additionally, diagnostic challenges exist because specialized testing and experienced clinicians are often required, which can create difficulties in rural or resource-limited settings. These factors may contribute to misdiagnosis or delayed diagnosis, potentially leading to more severe disease or poorer outcomes due to the postponement of appropriate treatment.

This case report describes a rare *Fusarium* infection in an otherwise immunocompetent patient, resulting in necrotizing myositis and periosteitis. We aim to highlight the importance of thorough investigation when confronted with challenging diagnostic cases and to stress that invasive fungal infections can occur in immunocompetent individuals, indicating that the threshold for testing should be increased when infections do not respond to standard antimicrobial therapy.

## Case presentation

The patient was a female in her 40s who initially presented to an outside emergency department with a three-to four-week history of a nonhealing wound over the right lower extremity following a dog bite. She had no known prior medical history aside from active tobacco use and a remote history of intravenous drug use. The animal was known to the patient and was confirmed to be up to date on all recommended veterinary vaccinations. Initial contrast-enhanced CT imaging demonstrated a 1.5 cm by 4.5 cm partially organized, heterogeneous fluid collection at the anterolateral distal lower extremity at the junction of the calf and ankle, suggestive of abscess or seroma. Additionally, there was soft tissue swelling extending to the periphery of the distal third of the anterior tibial bone, with disruption of the superficial fascial layers, myositis of the anterior muscle group, and herniation of the extensor hallucis longus muscle belly through the overlying wound defect.

Given the trauma from the dog bite, she was evaluated by trauma surgery, who recommended medical management only at that time because the abscess was considered less likely. The patient was discharged on oral doxycycline 100 mg every 12 hours for a seven-day course for presumptive treatment of simple cellulitis. She was seen at follow-up and prescribed an additional course of antibiotics, including amoxicillin-clavulanate 875 mg and trimethoprim-sulfamethoxazole one double-strength tablet every 12 hours, due to lack of improvement with the initial treatment, concern for progression of her condition, and the possibility of a polymicrobial infection involving anaerobic or resistant organisms.

Despite multiple courses of antibiotics, the patient’s condition worsened, with extension of the wounds that developed significant swelling and purulent drainage, prompting her to return to the emergency department two weeks later. On arrival, she was afebrile with a temperature of 97 °F and otherwise hemodynamically stable, with a blood pressure of 136/79 mmHg and a heart rate of 67 beats per minute. Physical examination revealed a distal right lower extremity wound with marked surrounding edema and copious purulent material extruding from the anterior tibial muscle group. She was clinically diagnosed at that time with a right lower extremity wound abscess. The wounds measured 5 x 2 x 0.5 cm and 3 x 3 x 0.5 cm.

Preliminary laboratory evaluation (Table 1) showed mildly elevated serum lactic acid at 2.37 mmol/L, with other parameters largely within normal limits. These included glucose at 118 mg/dL, sodium at 140 mEq/L, creatinine at 0.7 mg/dL, alkaline phosphatase (ALP) at 72 IU/L, alanine aminotransferase (ALT) at 22 IU/L, albumin at 3.0 g/dL, total protein at 6.5 g/dL, white blood cell count at 9.2 cells/mm³, platelets at 375 × 10⁹/L, and a negative HIV antigen/antibody test.

**Table 1:** Laboratory results

Laboratory test	Value	Units	Reference range
Serum lactic acid	2.37	mmol/L	0.5–2.2
Glucose	118	mg/dL	70–99
Sodium	140	mEq/L	135–145
Creatinine	0.7	mg/dL	0.6–1.2
Alkaline phosphatase (ALP)	72	IU/L	44–147
Alanine aminotransferase (ALT)	22	IU/L	7–56
Albumin	3	g/dL	3.5–5.0
Total protein	6.5	g/dL	6.0–8.3
White blood cell count	9.2	×10³ cells/mm³	4.5–11.0
Platelet count	375	×10⁹/L	150–450
HIV antigen/antibody test	Negative	—	Negative

**Figure 1:**
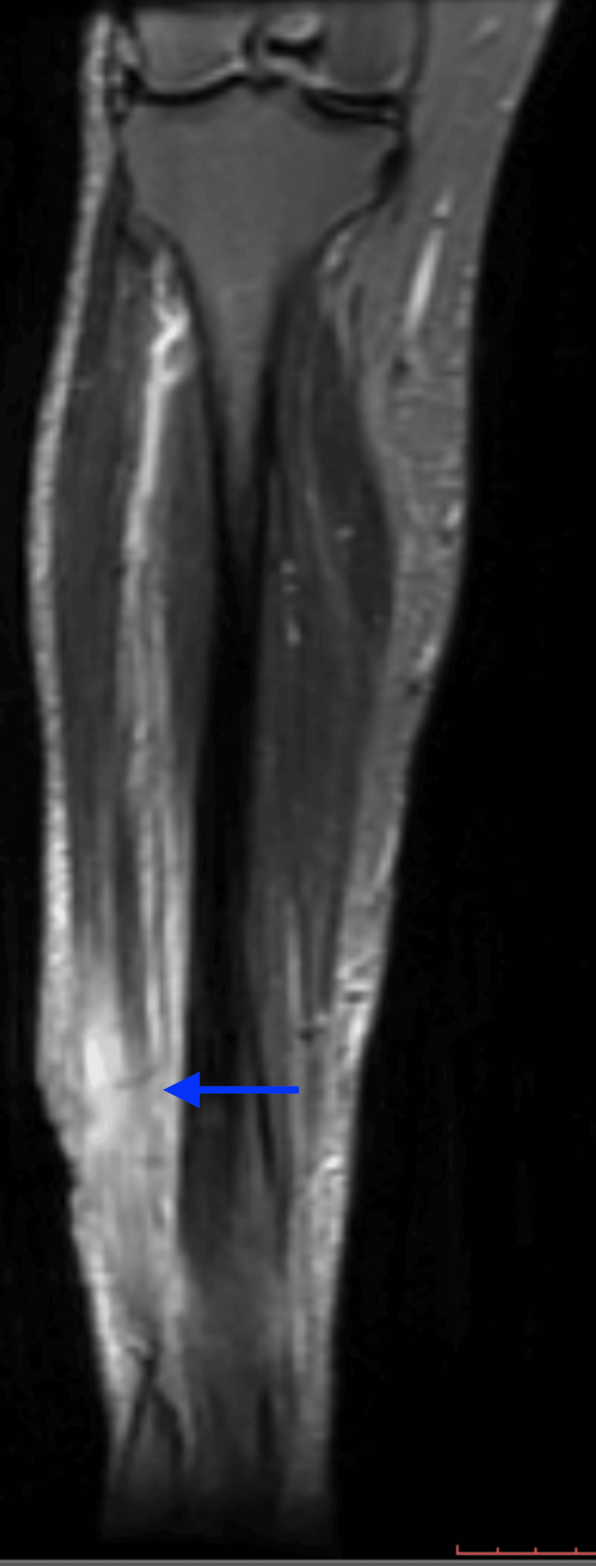
Coronal view of the T2-weighted MRI of the right lower extremity demonstrating area of inflammation and infection (blue arrow) MRI: magnetic resonance imaging

The patient successively underwent incision and drainage (I&D) with a Gram stain revealing 3+ (5-10/O.I.F) WBCs, 1+ Gram-positive cocci in pairs. Given the worsening condition on presentation during this hospitalization, a repeated CT was performed, which showed a large open wound along the anterolateral middle third portion of the right lower extremity with disruption of the fascial plane, profound muscle swelling, myositis, and herniation of the extensor hallucis muscle. A venous Doppler ultrasound was done concurrently, which did not show any evidence of deep vein thrombosis. Further investigation with MRI redemonstrated the large open wound and confirmed active myositis and involvement of the distal tibial periosteum on T2-weighted images (Figure 1).

**Figure 2:**
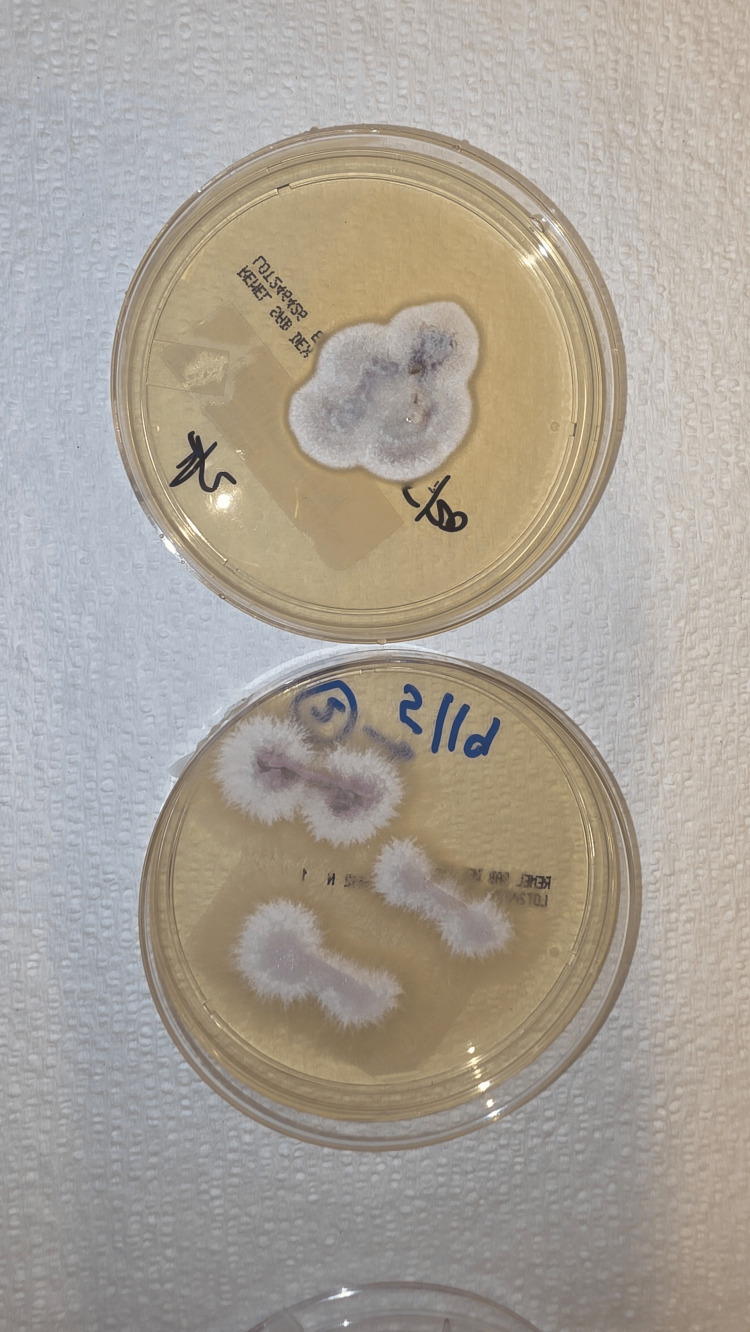
Macroscopic picture of Fusarium plated on agar with its characteristic woolly appearance and variable color (here appearing as light purple and white)

**Figure 3:**
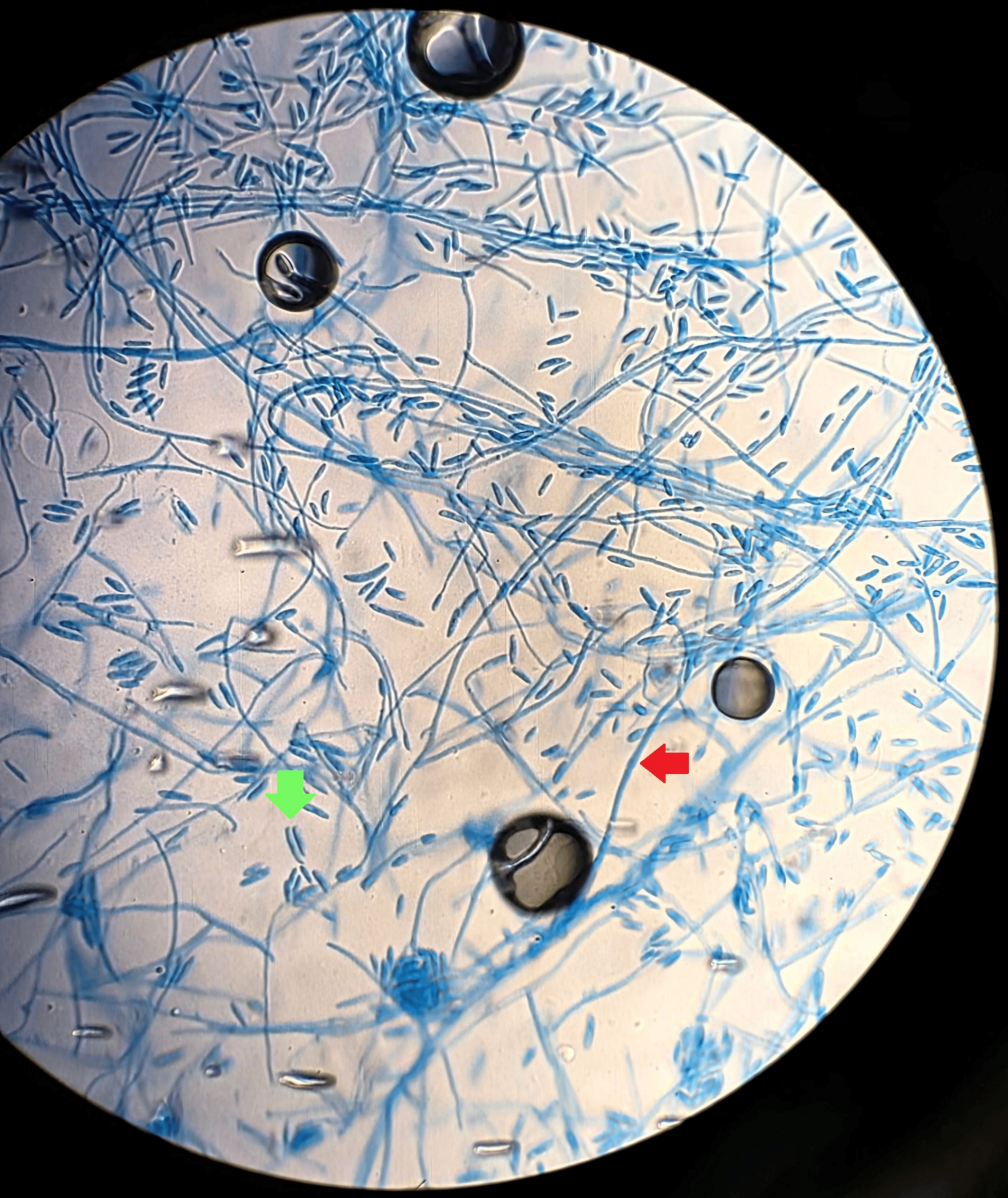
Microscopic view of Fusarium with characteristic filamentous septations (red arrow) and conidia (green arrow)

Cultures from the samples subsequently grew a few colonies of methicillin-resistant *Staphylococci* (MRSA), rare growth of *Stenotrophomonas* *maltophilia,* and a few colonies of mold on Sabouraud dextrose agar (SDA). The later cultures eventually grew out to demonstrate white, fluffy, septated hyphae molds which were identified as *Fusarium *(Figures 2-3). The bacterial wound cultures showed 1+ growth of MRSA with a minimum inhibitory concentration (MIC) for vancomycin of 1.0 (S), tetracycline <=1 (S), levofloxacin (R), sulfamethoxazole-trimethoprim >=320 (R), and *Stenotrophomonas* with MIC. Levofloxacin 1 (S). The microbiology laboratory was unable to speciate *Fusarium*. Multiple sets of blood cultures remained negative, with a transthoracic echocardiogram obtained showing no evidence of valvular vegetation. 

Due to findings of extensive tissue injury and involvement on imaging, the patient underwent further I&D and debridement and was noted intraoperatively to have gross evidence of purulence, muscle necrosis, and edematous soft tissues. Intraoperative cultures and histopathology were submitted, and the patient was discharged on oral doxycycline 100 mg every 12 hours along with oral metronidazole 500 mg every eight hours while microbiology and pathology reports pended. She later returned to the emergency department and was reassessed by trauma surgery, who proceeded with another intraoperative evaluation and debridement, noting extensive eschar and necrotic soft tissue and muscle. No cultures were obtained at this procedure, but a wound vacuum-assisted closure (VAC) device was applied.

**Figure 4:**
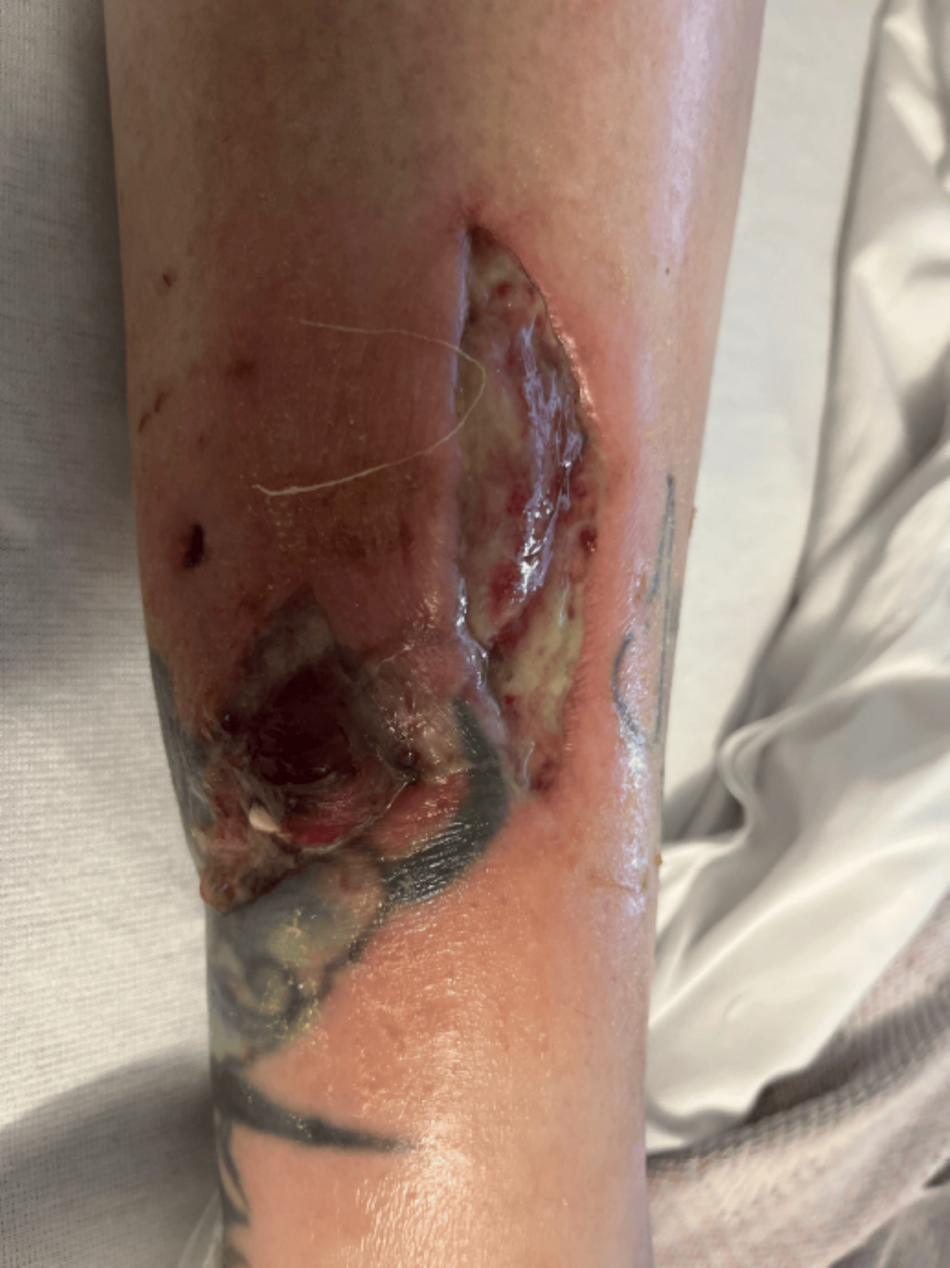
The wound post-debridement with improving erythema and purulence after the initiation of antifungal therapy

**Figure 5:**
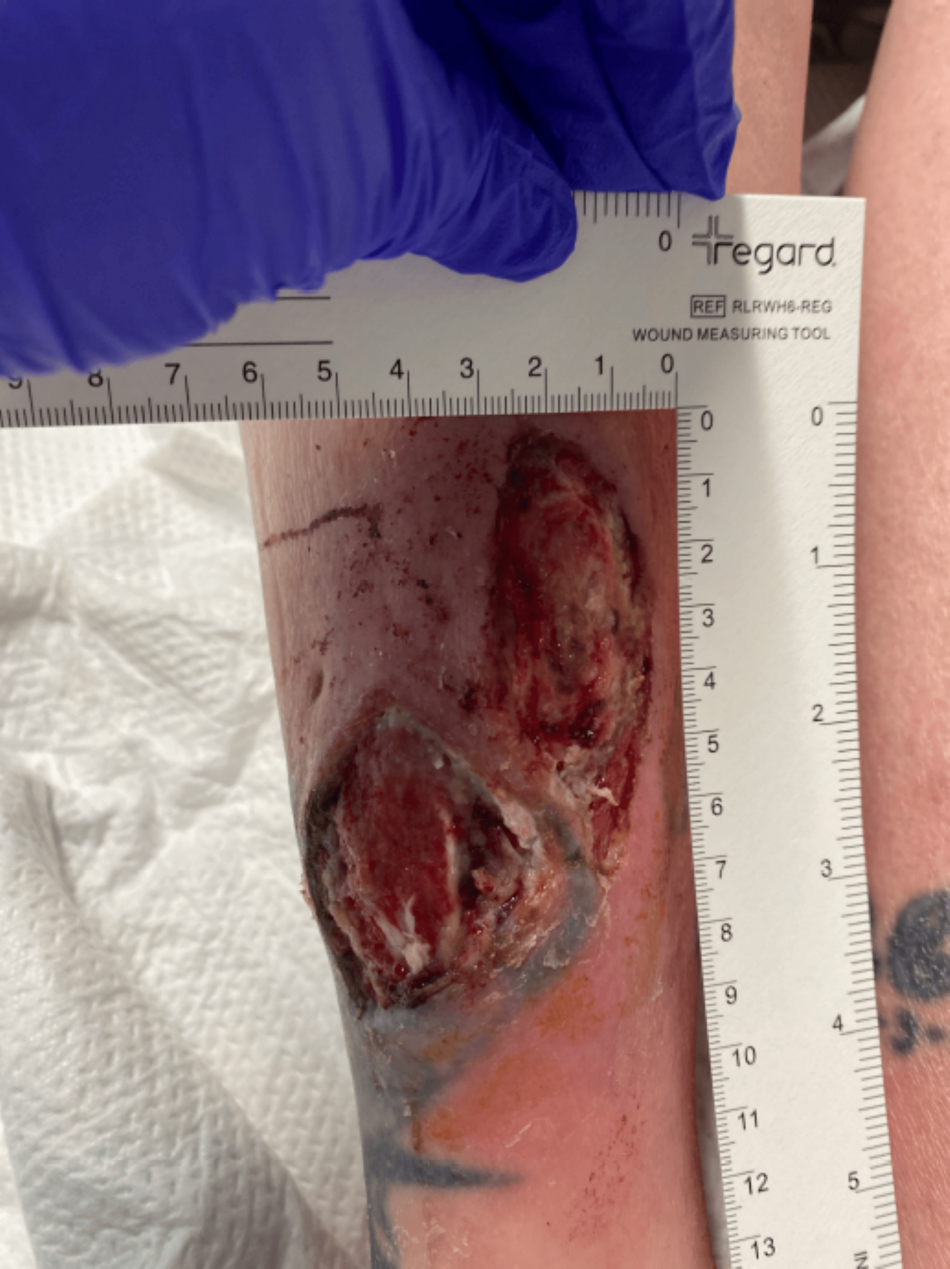
The wound on initial clinical follow-up

**Figure 6:**
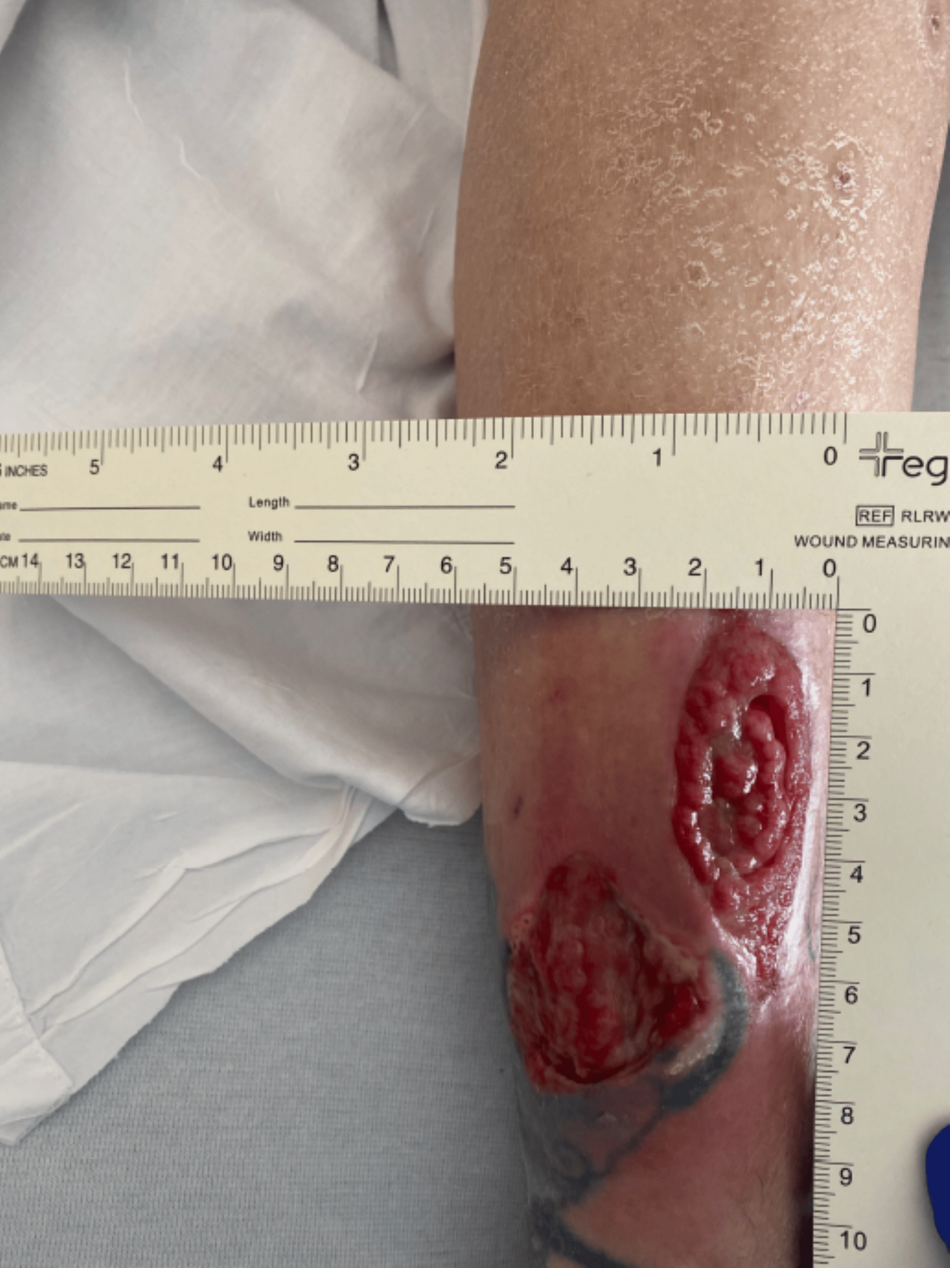
The wound on subsequenta clinical follow-up with resolving infection and development of healthy granulation tissue

Postoperatively, the patient was started on intravenous antibiotics, including vancomycin 15 mg/kg every 24 hours based on trough levels, sulfamethoxazole-trimethoprim 800/160 mg every 12 hours, and levofloxacin 750 mg daily. As the infection did not improve and there was concern for worsening edema and purulence, antimicrobial coverage was broadened to include antifungal therapy with liposomal amphotericin B 5 mg/kg IV every 24 hours and voriconazole 200 mg IV every 12 hours, and she was switched to IV daptomycin 8 mg/kg every 24 hours. After 14 days of treatment, there was marked improvement in wound size, drainage, and swelling (Figure 6). The patient was then cleared for transition to oral antimicrobials at discharge, with a regimen of voriconazole, levofloxacin, and doxycycline. Outpatient follow-up demonstrated continued significant wound healing over the following weeks (Figures 4-6), and a six- to eight-week course of oral therapy was planned post-discharge.

## Discussion

While systemic invasive *Fusarium* infections have been widely reported in immunocompromised individuals, particularly in patients with hematologic malignancies [1-4], cases in immunocompetent patients are rare. Among invasive mold infections (IMDs), *Fusarium* is the most common, accounting for approximately 36% of all IMDs, and carries the highest mortality rate, followed by *Scedosporium* at 14% in patients with allogeneic HSCT (alloHSCT) and hematologic malignancies. Data regarding IMDs, and specifically *Fusarium* infections, in non-immunocompromised patients remain limited [15]. Among the few reported cases in immunocompetent individuals, most had underlying comorbidities, including diabetes [6-7], trauma [8-9], cardiovascular disease [6,13], chronic obstructive pulmonary disease, asthma, or extensive burns [14]. In these patients, infections were most commonly limited to superficial skin involvement [1].

Osteomyelitis of non-immunocompromised individuals is rare but has been described in connection with direct exposure to soil in an agricultural laborer [8], by way of water contaminated with soil [16], and also as a postoperative complication [17]. To the authors' knowledge, there has only been one reported case in the related literature in English of visceral disseminated *Fusarium solani* in a non-immunocompromised adult male [10]. While not considered an immunocompromising condition traditionally by many, the presence of diabetes, especially that which is uncontrolled, has been identified as a risk factor for suppressing immune function and risk of infection by emerging molds such as *Fusarium*, *Scedosporium*, and *Mucorales *[18-20].

Antifungal azoles, particularly voriconazole, have been widely used as first-line therapy for systemic fungal infections [10-14], with reported success rates of over 50% [10]. Because *Fusarium* susceptibility in vivo is difficult to predict, the standard approach is combination therapy with voriconazole and liposomal amphotericin B until culture susceptibilities are available [13], as was implemented in our patient. In this case, it took nine days from initial presentation to identify *Fusarium* isolates. Although studies indicate that all currently available systemic antifungal agents show some activity against various *Fusarium* species, the level of activity is often low, whereas amphotericin demonstrates the highest efficacy, supporting its use as a first-line agent.

Our case remains unique in that the patient was a young individual without predisposing medical conditions such as malignancy or diabetes. While the patient was initially treated with broad-spectrum anti-infectives targeting bacterial causes, operative cultures revealed MRSA and *Stenotrophomonas maltophilia*, though growth was sparse, described as 1+, and observed only on Gram stains. Moreover, the patient's infection did not respond to the initial course of conventional antibiotics directed at MRSA and actually worsened during treatment, but improved after the addition of amphotericin and voriconazole. Given cthe linical response to anti-fungal therapy and the growth of *Fusarium* from tissue culture, it is highly suggestive that the true pathogen was the *Fusarium* mold while MRSA and Stenotrophomonas were likely colonizers. Regretfully, the microbiology laboratory discarded the specimen, and so speciation and antifungal sensitivity panel were not performed. Nevertheless, the patient made significant clinical recovery with a combination of voriconazole and liposomal amphotericin B therapy (Figures 1-3) and continues to do well in follow-up.

## Conclusions

*Fusarium* infections are rare causes of invasive disease in humans and primarily affect individuals with predisposing immunosuppressive conditions, including those receiving immunosuppressive therapy, with hematologic malignancies, after alloHSCT, or with underlying medical conditions such as diabetes or chronic lung disease. Reports of *Fusarium* infection in patients without predisposing factors remain uncommon and are more often limited rather than invasive. Because most existing literature focuses on predisposing factors and management of invasive fungal infections in immunocompromised patients, further research is needed to clarify the factors that contribute to infection in immunocompetent individuals, to better define risk, and to determine optimal management strategies. By highlighting these unusual cases, we aim to advance understanding of the disease process and promote earlier recognition of patients who require treatment.
